# GPC2 promotes prostate cancer progression via MDK-mediated activation of PI3K/AKT signaling pathway

**DOI:** 10.1007/s10142-024-01406-y

**Published:** 2024-07-17

**Authors:** Sijin Chen, Jiaxing Liao, Juhua Li, Saihui Wang

**Affiliations:** Department of Urology, Hunan Provincial People’s Hospital, The First Affiliated Hospital of Hunan Normal University, Changsha, 410005 Hunan Province China

**Keywords:** GPC2, Prostate cancer, PI3K/AKT signaling, MDK

## Abstract

Prostate cancer is a major medical problem for men worldwide. Advanced prostate cancer is currently incurable. Recently, much attention was paid to the role of GPC2 in the field of oncology. Nevertheless, there have been no investigations of GPC2 and its regulatory mechanism in prostate cancer. Here, we revealed a novel action of GPC2 and a tumor promoting mechanism in prostate cancer. GPC2 was upregulated in prostate cancer tissues and cell lines. Higher expression of GPC2 was correlated with higher Gleason score, lymphatic metastasis, and worse overall survival in prostate cancer patients. Decreased expression of GPC2 inhibited cell proliferation, migration, and invasion in prostate cancer, whereas GPC2 overexpression promoted these properties. Mechanistically, GPC2 promoted the activation of PI3K/AKT signaling pathway through MDK. The rescue assay results in prostate cancer cells demonstrated that overexpression of MDK could attenuate GPC2 knockdown induced inactivation of PI3K/AKT signaling and partly reverse GPC2 knockdown induced inhibition of cell proliferation, migration, and invasion. In all, our study identified GPC2 as an oncogene in prostate cancer. GPC2 promoted prostate cancer cell proliferation, migration, and invasion via MDK-mediated activation of PI3K/AKT signaling pathway. GPC2 might be a promising prognosis predictor and potential therapeutic target in prostate cancer.

**Supplementary Information:**

The online version contains supplementary material available at 10.1007/s10142-024-01406-y

## Introduction

Prostate cancer (PC) is the most prevalently diagnosed non-cutaneous malignancy in male patients, with a reported over 190,000 new cases in the United States in 2020(Desai et al. 2021). It is also the second leading cause of cancer-related death in men and about 33,330 deaths were estimated in US in 2020(.Siegel et al. 2020; Yamada and Beltran 2021). Though the incidence of PC is lower in China than that in US, it represents a major medical problem for men and is nonetheless increasing every year. Current treatment strategies for PC patients include surgery, androgen deprivation therapy (ADT), radiation, chemotherapy, targeted therapy, immunotherapy and so on(.Rebello et al. 2021; Sekhoacha et al. 2022). Though these methods exhibited promising efficiency for localized disease, the prognosis for advanced prostate cancer is still unsatisfactory(.Haffner et al. 2021). Moreover, due to the lack of methods for early clinical diagnosis, about 15% of patients had regional disease that has spread to regional lymph nodes, and 5% of cases had distant metastases(.Wang et al. 2018; Wasim et al. 2022). The unsatisfactory outcome of the present treatment for middle- or late-stage of the disease limits the overall survival of prostate cancer patients, thus an improved therapeutic approach is warrant needed. Exploring novel driver genes participating in the initiation and progression of prostate cancer is of great value for the identification of potential therapeutic targets.

*GPC2* belongs to the glypican (GPC) family genes that contains six members, including *GPC1*, *GPC2*, *GPC3*, *GPC4*, *GPC5*, and *GPC6*, in mammalian genome(.Li et al. 2018). In general, glypican proteins were located in the cellular membrane by the means of a glycosylphosphatidylinositol (GPI) anchor, and mainly functioned as a protein co-receptor and participated in intracellular signal transduction(.Kaur and Cummings 2019). Previous studies manifest that these glypicans were critical in regulating multiple diverse cellular functions, including cell survival, differentiation, and cell morphology(.Filmus 2022; Filmus and Capurro 2014; Li et al. 2020). GPC2, a heparan sulfate proteoglycan oncoprotein, had been shown to aberrantly expressed in multiple types of tumors and connected with the tumorigenesis and development(.Bosse et al. 2017; Chen et al. 2022; Lin et al. 2022; Raman et al. 2021). Moreover, researches showed that enforced expression of GPC2 contributed to malignant behaviors of cancer cells and might act as potential cancer therapeutic targets. Whereas, to date, the GPC2 role in prostate cancer remains unreported.

In the present study, we demonstrated that GPC2 was an oncogene in prostate cancer. Through data mining using online tool and bioinformatic methods, we found the GPC2 was upregulated in prostate cancers and its higher expression predicted poor patients’ prognosis. In *vitro* experiments revealed that overexpression of GPC2 promoted cell proliferation, migration, and invasion in prostate cancer cells and silence of GPC2 caused the opposite effects. Functional enrichment analysis suggested that GPC2-related differentially expressed genes (DEGs) were mainly enriched in PI3K/AKT signaling pathway. Further experiments confirmed that GPC2 positively regulated PI3K/AKT signaling though MDK, and overexpression of MDK attenuated the inhibitory effect of GPC2 knockdown on malignant behaviors of prostate cancer. Our study provides a scientific basis for finding novel target therapy methods in prostate cancer and the GPC2/MDK/PI3K/AKT signaling axis might be promising therapeutic target.

## Materials and methods

### Cell culture

Human normal (RWPE-1) and cancerous prostate epithelial cell lines including DU145, PC-3, and LNCap were all collected from the Cell Bank of Type Culture Collection (CBTCC, Chinese Academy of Sciences, Shanghai, China). RWPE-1 were maintained in Keratinocyte-SFM medium. The prostate cancer cell lines including DU145, PC-3, and LNCap were cultured in DMEM, F-12 K, and RPMI 1640 mediums. All the mediums were supplemented with 10% fetal bovine serum (FBS; Gibco, USA) and 1% penicillin/streptomycin. Cells were cultured in a humidified incubator at 37 °C with an atmosphere of 5% CO2.

### Plasmid transfection

MDK overexpression vector (pcDNA3.1-MDK-Flag) and empty vector were purchased from Sangon Biotech (Shanghai, China). For plasmid transfection, cells were seeded into 6-well plate and cultured to 60–80% confluence. 200 µL Opti-MEM containing 5 µL HighGene reagent were mixed with 3 µg plasmid, and further incubated for 5 min at room temperature. Then, the mixture was added into each well of the plate. The cells were cultured for another 12 h and washed with PBS twice and replaced with fresh complete medium. 48 h after transfection, cells were harvest, then qRT-PCR and western blot assays were performed to assess the transfection efficiency.

### Lentivirus infection

Lentivirus containing GPC2 overexpression vector (LV-GPC2) or empty vector (LV-Ctrl), and shRNA targeting GPC2 (shGPC2, 5’-GATCCGTTTGATGTACCTGCAGGAAACTCGAGTTTCCTGCAGGTACATCAAACTTTTTG-3’) or the negative control shRNA (shNC) were purchased from Hanbio (Shanghai, China). The lentivirus infection assay was conducted according to the manufacturer’s instruction. Stably infected cells were selected using puromycin (5 µg/µL). The knockdown or overexpression of GPC2 in stably infected cell lines were demonstrated by qRT-PCR and western blot assays.

### CCK-8 assay

The DU145 and PC-3 cells (5 × 10^3^) in 100 µL complete medium were seeded into each well of 96-well plates and cultured for 0, 24, 48, and 72 h. Then, 10 µL CCK-8 reagent (Dojindo, Tokyo, Japan) was added to each well and further incubated for 2 h at 37 °C in dark. The absorbance at 450 nm was measured using a microplate reader (Thermo Fisher Scientific, USA).

### Colony formation assay

The DU145 and PC-3 cells were digested and resuspended in complete medium at a density of 5 × 10^4^/ml. A total of 5 × 10^2^ cells were seeded into each well of the 6-well plate. The plate was maintained in the humidified incubator at 37 °C with 5% CO2. The supernatant was removed and replaced with fresh complete medium every five days. After two weeks, the cells were washed with ice-cold PBS and fixed with 4% paraformaldehyde for 10 min at room temperature. The cells were then stained with crystal violet, and the colonies were photographed. The number of colonies in each well were counted using ImageJ software.

### Wound-healing assay

The DU145 and PC-3 cells in the logarithmic growth phase were seeded in 6-well plates and cultured in complete medium. When the cell density reached 100%, equidistant scratches in the bottom of 6-well plates were made with a sterile pipette tip. After washing with cold PBS once, the prime image was immediately photographed. Then, the cells were cultured in serum-free medium for another 24 h, the wound were observed under the inverted microscope and photographed. Would healing rate was determined using the ImageJ software.

### Transwell invasion assay

The DU145 and PC-3 cells were digested with calcium-free trypsin and were resuspended in medium without FBS at a density of 5 × 10^4^/ml. 200 µl of cell suspension were added into the upper of the transwell chamber that was precoated with Matrigel (Corning, USA). The bottom chamber was added with 600 µl of medium containing 20% FBS. After incubation for 24 h in the standard circumstance, the medium was discarded. The invaded cells on the lower surface of the chamber were fixed with 4% paraformaldehyde, and were stained with crystal violet. After being washed with PBS twice, the invaded cells in each chamber were observed in five randomly selected fields and photographed using the inverted microscope, and cells were counted using the ImageJ software.

### RNA isolation and qRT-PCR

Total RNA was isolated from tissues and cells using TRNzol universal reagent kit (TIANGEN Biotech, China) according to the manufacturer’s instructions, and were then reverse transcribed into cDNA using the RevertAid First Strand cDNA Synthesis Kit (Thermo Fisher Scientific, Waltham, MA, USA). Quantitative real-time PCR (qRT-PCR) was conducted using Talent qPCR Premix (SYBR Green) (TIANGEN Biotech, China). Relative mRNA expression levels of target genes were calculated by the 2^−ΔΔCt^ method and the *GAPDH* expression was served as internal control. The primers sequence used in the qRT-PCR assay were as follow: *GPC2*, forward 5’-GACTACCTGCTCTGCCTCTC-3’ and reverse 5’-GAACCACGTTGAGGCAGAAG-3’; *MDK*, forward 5’-GATAAGGTGAAGAAGGGCGG-3’ and reverse 5’-TCGGCTCCAAACTCCTTCTT-3’; *GAPDH*, forward 5’-CCATCTTCCAGGAGCGAGAT-3’ and reverse 5’-TGAGTCCTTCCACGATACCA-3’.

### **Western blot and** immunoprecipitation **assays**

The total protein content was extracted from DU145 and PC-3 cells using RIPA lysis buffer (Solarbio, Beijing, China) containing 1% protease inhibitor (Beyotime, China). After measuring the concentration of the total protein, 15 µg of protein was subjected to SDS-PAGE electrophoresis at 80 V for 2 h, and the separated protein was then transferred into the PVDF membranes. Subsequently, the membranes were blocked in 5% nonfat dry milk at room temperature for 1 h. After being washed with TBST three times, the membranes were incubated with the primary antibodies at 4 °C overnight. The next day, the membranes were washed with TBST three times and incubated with the specified secondary antibodies at room temperature for 1 h. The membranes were incubated with the enhanced chemiluminescence reagent for 30s, and then, protein bands were visualized using the Bio-Rad gel imaging system. The protein expression of GAPDH was regarded as internal control, and the relative protein levels were determined using the ImageJ software. For immunoprecipitation (IP) assay, total proteins were isolated using IP lysis buffer were immunoprecipitated using GPC-2 antibody or IgG at 4 °C overnight. The next day, the immunoprecipitated proteins were incubated with protein A/G-Sepharose beads for 2 h at 4 °C. Then, the immunoprecipitated proteins were washed with cold phosphate-buffered saline (PBS) three times, and were finally suspended in 30 µl of 1× loading buffer for Western blot.

### Bioinformatic analysis

TIMER (https://cistrome.shinyapps.io/timer/) online tool was employed to analyzed the expression profile of GPC2 across tumors. UALCAN database (http://ualcan.path.uab.edu/) was used to compare the expression of GPC2 in prostate cancer tissues and adjacent normal tissues, and to explore the correlation of GPC2 expression and patients’ Gleason score and lymphatic metastasis. GEPIA (http://gepia.cancer-pku.cn/) web tool was utilized to investigate the association of GPC2 expression and patients’ overall survival. The expression data of prostate carcinoma in TCGA database was separated into GPC2^High^ and GPC2^Low^ groups, and differentially expressed genes (DEGs) between the two groups were identified using *egdeR* package in R. GO and KEGG enrichment analyses were performed using DAVID (Database for Annotation, Visualization and Integrated Discovery) database (https://david.ncifcrf.gov/). STRING database (https://string-db.org/) was utilized to construct a protein-protein interaction (PPI) network based on the DEGs.

### Sample collection and immunohistochemistry staining

Ten paired PCa tissues and adjacent normal tissues were obtained from patients who underwent surgery at the Department of Urology, Hunan Provincial People’s Hospital. The detailed information of the ten patients was shown in Supplementary Table S1. This study was approval by the Research Ethics Committee of Hunan Provincial People’s Hospital. Samples were fixed in 4% paraformaldehyde after resection. All samples were collected after obtaining the signed consent of patients. After being embedded in paraffin, samples were sliced into 4-µm slices. Subsequently, the slices were deparaffinized in xylene and dehydration, followed by blocking with 3.0% hydrogen peroxide. Then, the slides were incubated with primary antibodies against GPC2 (1:200, sc-393,824, Santa Cruz Biotechnology) overnight at 4 °C. The next day, the slides were incubated with HRP-conjugated goat anti-rabbit IgG at room temperature. The reaction was then performed using a DAB reagent, followed by hematoxylin stain for 45 s as a counterstain. Finally, the slides were observed and photographed under a microscope by two independent pathologists. The percentage of positive staining cells was quantified using Image J software.

### Statistical analysis

All the statistical analyses were conducted using R and GraphPad Prism 8.0 software. Data were shown as Mean ± SD (standard deviation). Differences between groups were compared using Student’s *t*-test or one-way ANOVA, and *P* value less than 0.05 was regarded as statistically significant.

## Results

### The expression of GPC2 was upregulated in prostate cancer and was negatively correlated with patients’ prognosis

**Fig. 1:**
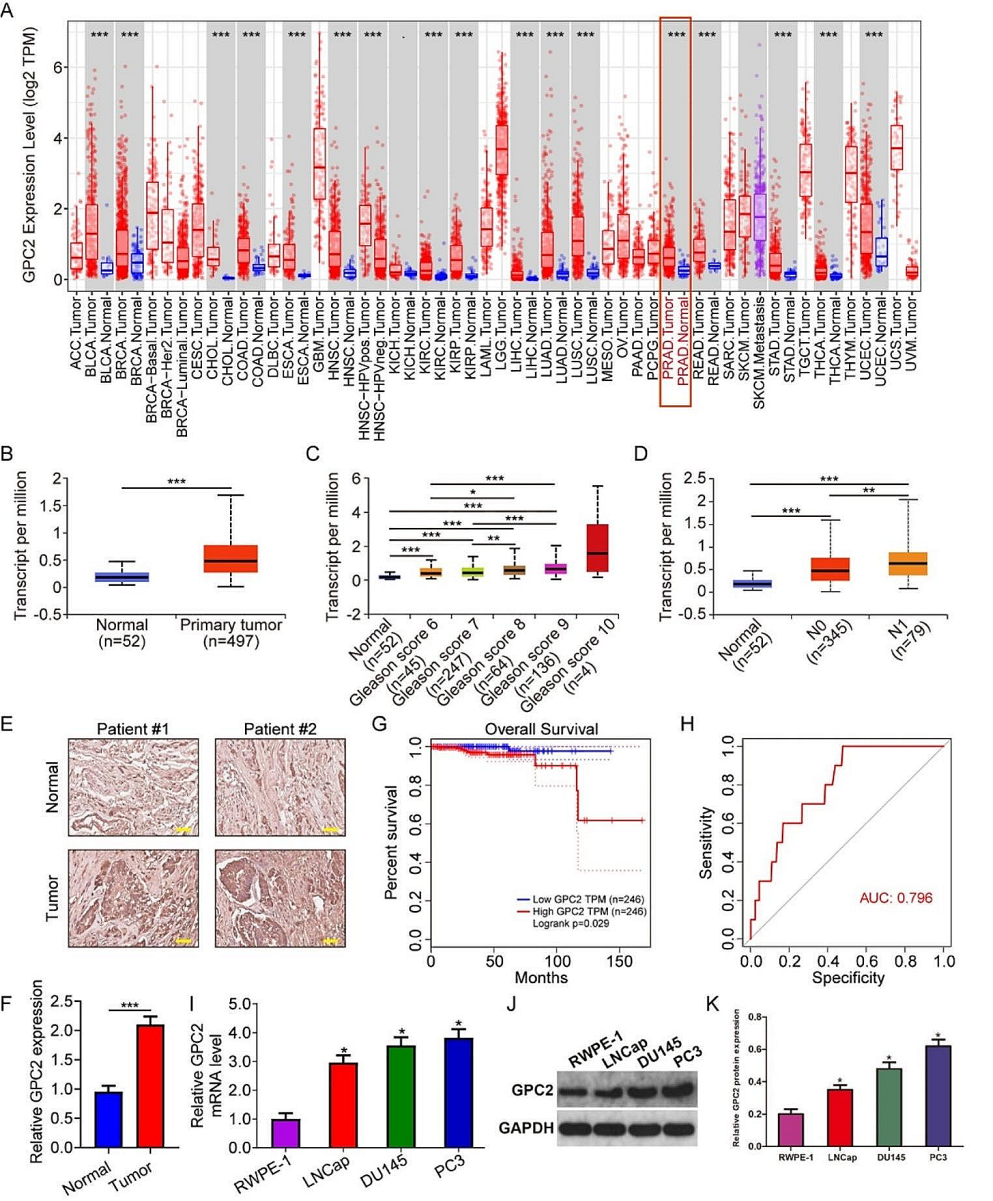
The expression of GPC2 was upregulated in prostate cancer and was negatively correlated with patients’ prognosis. (**A**) The expression level of GPC2 across various tumor types. ACC, adrenocortical carcinoma; BLCA, bladder urothelial carcinoma; BRCA, breast invasive carcinoma; CESC, cervical squamous cell carcinoma; CHOL, cholangiocarcinoma; COAD, colon adenocarcinoma; DLBC, diffuse large B-cell lymphoma; ESCA, esophageal carcinoma; GBM, glioblastoma multiforme; HNSC, head and neck squamous cell carcinoma; KICH, kidney chromophobe; KIRC, kidney renal clear cell carcinoma; KIRP, kidney renal papillary cell carcinoma; LAML, acute myeloid leukemia; LGG, brain lower grade glioma; LIHC, liver hepatocellular carcinoma; LUAD, lung adenocarcinoma; LUSC, lung squamous cell carcinoma; MESO, mesothelioma; OV, ovarian serous cystadenocarcinoma; PAAD, pancreatic adenocarcinoma; PCPG, pheochromocytoma and paraganglioma; PRAD, prostate adenocarcinoma; READ, rectum adenocarcinoma; SARC, sarcoma; SKCM, skin cutaneous melanoma; STAD, stomach adenocarcinoma; TGCT, testicular germ cell tumors; THCA, thyroid carcinoma; THYM, thymoma; UCEC, uterine corpus endometrial carcinoma; UCS, uterine carcinosarcoma; UVM, uveal melanoma. (**B**) Comparison of GPC2 expression level in prostate cancer tissues and adjacent normal tissues. (**C**) Higher expression of GPC2 was correlated with increased Gleason score. (**D**) Higher expression of GPC2 was correlated with lymphatic metastasis. (**E-F**) IHC analysis of GPC2 expression in normal and tumor tissues, and quantitative analysis. Scale bar, 200 µM. (**G**) Kaplan-Meier survival analysis and Log-rank test revealed that the higher GPC2 expression predicted worse overall survival. (**H**) ROC curve analysis of the predictive performance of GPC2 expression level in prostate cancer. (**I-K**) The mRNA and protein expression level of GPC2 in normal (RWPE-1) and cancerous prostate epithelial cell lines including DU145, PC-3, and LNCap. *P < 0.05, ***P < 0.001

We first detected the expression of GPC2 in different types of tumors based on TIMER database and the results revealed that GPC2 was consistently upregulated in multiple tumors including bladder urothelial carcinoma (BLCA), breast invasive carcinoma (BRCA), cholangiocarcinoma (CHOL), colon adenocarcinoma (COAD), esophageal carcinoma (ESCA), head and neck squamous cell carcinoma (HNSC), kidney renal clear cell carcinoma (KIRC), kidney renal papillary cell carcinoma (KIRP), kidney chromophobe (KICH), liver hepatocellular carcinoma (LIHC), lung adenocarcinoma (LUAD), lung squamous cell carcinoma (LUSC), prostate adenocarcinoma (PRAD), rectum adenocarcinoma (READ), stomach adenocarcinoma (STAD), thyroid carcinoma (THCA), as well as uterine corpus endometrial carcinoma (UCEC) (Fig. 1A), suggesting the potential role of GPC2 in tumorigenesis and progression. Then, using the UALCAN database, we also found a significant higher expression of GPC2 in prostate cancer tissues compared to that in normal tissues (Fig. 1B). Moreover, GPC2 expression was markedly increased with the increase of patients’ Gleason score and lymphatic metastasis (Fig. 1C-D). IHC analysis also confirmed that the protein level of GPC2 was higher in prostate cancer tissues than that in normal tissue (Fig. 1E-F). Besides, we performed Kaplan-Meier survival analysis based on the TCGA database data and results revealed that the high GPC2 expression group had a poorer overall survival than that of the low GPC2 expression group (Fig. 1G). ROC curve analysis showed that the AUC value was 0.796, suggesting GPC2 might be served as a biomarker to predict prostate cancer prognosis (Fig. 1H). Finally, we examined the abundance of GPC2 in both normal (RWPE-1) and cancerous prostate epithelial cell lines including DU145, PC-3, and LNCap by qRT-PCR. The results showed that the mRNA and protein levels of GPC2 was significantly higher in the DU145 and PC-3 cell line in comparison to RWPE-1 (Fig. 1I-K).

### Silence of GPC2 inhibited cell proliferation, migration, and invasion in prostate cancer cells

**Fig. 2:**
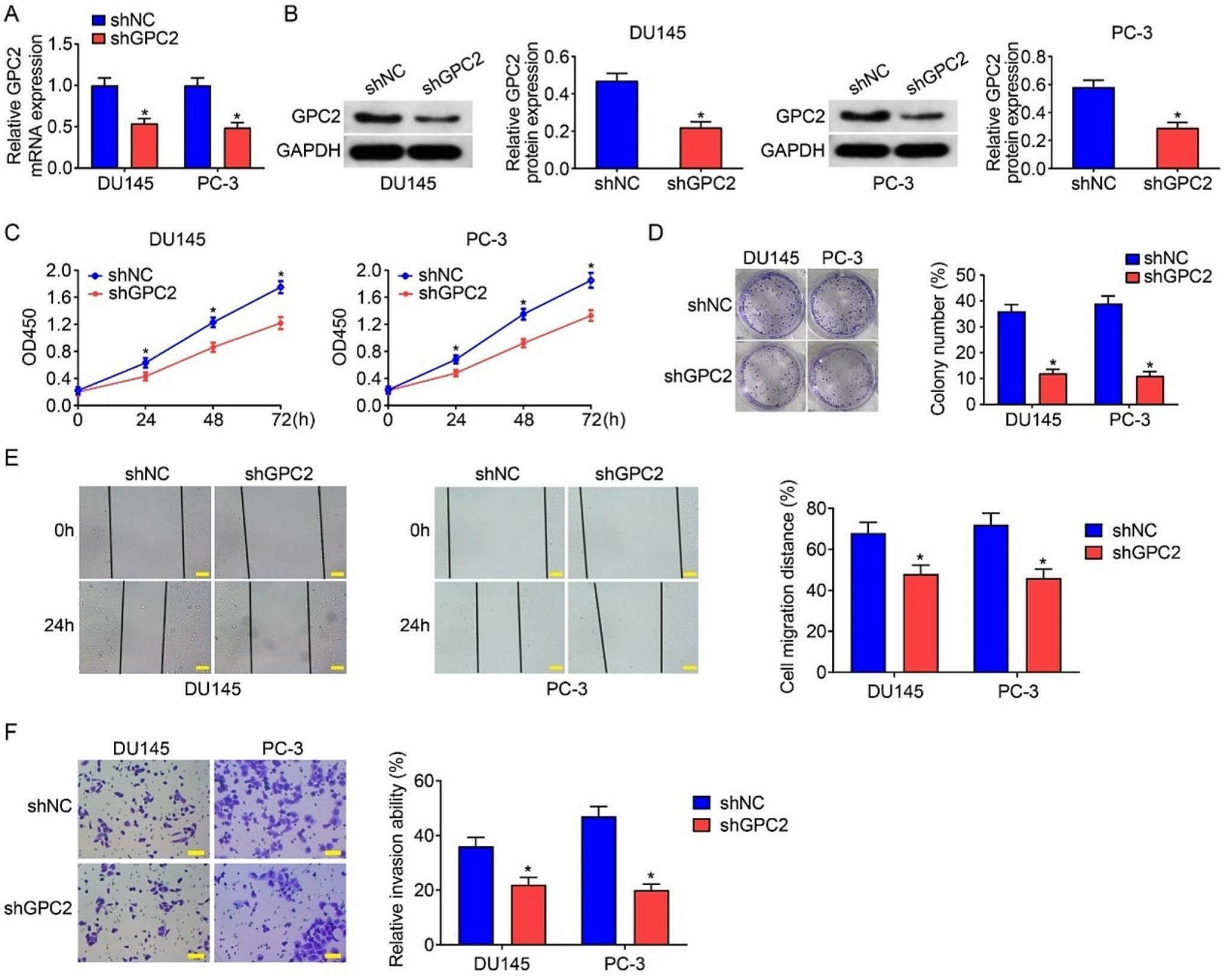
Silence of GPC2 inhibited cell proliferation, migration, and invasion in prostate cancer cells. (**A**) qRT-PCR assay confirmed the knockdown efficiency of shRNA targeting GPC2. (**B**) Western blot assay examined the protein level of GPC2 in shNC and shGPC2 groups and quantitative analyses. (**C-D**) CCK-8 and colony formation assays showed decreased proliferation ability after silencing GPC2. (**E**) Wound healing assay revealed that silence of GPC2 inhibited cell migration. Scale bar, 100 μm. (**F**) Transwell invasion assay revealed that silence of GPC2 inhibited cell invasion. Scale bar, 200 μm. Differences between groups were compared using Student’s *t*-test, *P < 0.05

To further explore the function of GPC2, we first silenced the expression of GPC2 in prostate cancer cells (DU145 and PC-3) through lentivirus infection and performed a series of function studies. The knock down efficiency of the specific shRNA targeting GPC2 (shGPC2) was verified both in mRNA and protein levels (Fig. 2A-B). CCK-8 and colony formation assays suggested that silence of GPC2 was remarkably able to inhibit the cell proliferation in DU145 and PC-3 cells (Fig. 2C-D). Additionally, wound healing and transwell invasion assays revealed that when the expression of GPC2 was knocked down, the migration and invasion abilities of DU145 and PC-3 cells were suppressed (Fig. 2E-F). Taken together, these results indicated that silence of GPC2 depleted the malignancy of prostate cancer cells.

### Overexpression of GPC2 promoted cell proliferation, migration, and invasion in prostate cancer cells

**Fig. 3:**
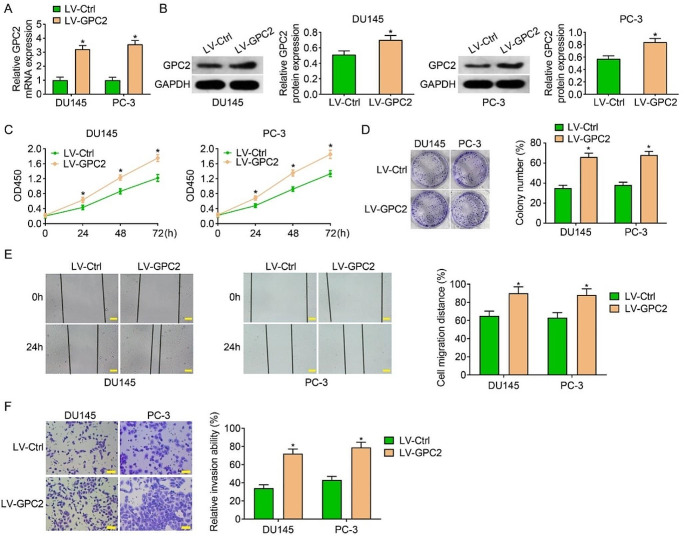
Overexpression of GPC2 promoted cell proliferation, migration, and invasion in prostate cancer cells. (**A**) qRT-PCR assay showed the efficiency of overexpressing GPC2 in DU145 and PC-3 cells. (**B**) Western blot assay examined the protein level of GPC2 in LV-Ctrl and LV-GPC2 groups and quantitative analyses. (**C-D**) CCK-8 and colony formation assays showed increased proliferation ability after overexpressing GPC2. (**E**) Wound healing assay revealed that overexpression of GPC2 promoted cell migration. Scale bar, 100 μm. (**F**) Transwell invasion assays revealed that overexpression of GPC2 promoted cell invasion. Scale bar, 200 μm. Differences between groups were compared using Student’s *t*-test, *P < 0.05. *P < 0.05

We also explored the effect of GPC2 overexpression on the malignancy of prostate cancer cells. As shown in Fig. 3A-B, qRT-PCR and western blot results suggested that the lentiviral over-expression vector (LV-GPC2) could boost both the mRNA and protein levels in DU145 and PC-3 cells. We then tested the proliferation ability though CCK-8 and colony formation assays, and the results showed the OD values and number of colonies were higher in LV-GPC2 group than that in LV-Ctrl group, indicating the proliferation of DU145 and PC-3 cells was accelerated due to the over-expression of GPC2 (Fig. 3C-D). In the wound healing assay, the healing rate was increased in LV-GPC2 group than that in LV-Ctrl group, revealing cell migration was promoted when GPC2 was upregulated (Fig. 3E). Similarly, markedly increased cell invasion ability was observed in DU145 and PC-3 cells stably overexpression of GPC2 (Fig. 3F). Thus, our loss-and-gain of function experiments illustrated that GPC2 promoted prostate cancer cell proliferation, migration, and invasion in *vitro*.

### Bioinformatic analysis identified GPC2-related DEGs and functional enrichment analysis

**Fig. 4:**
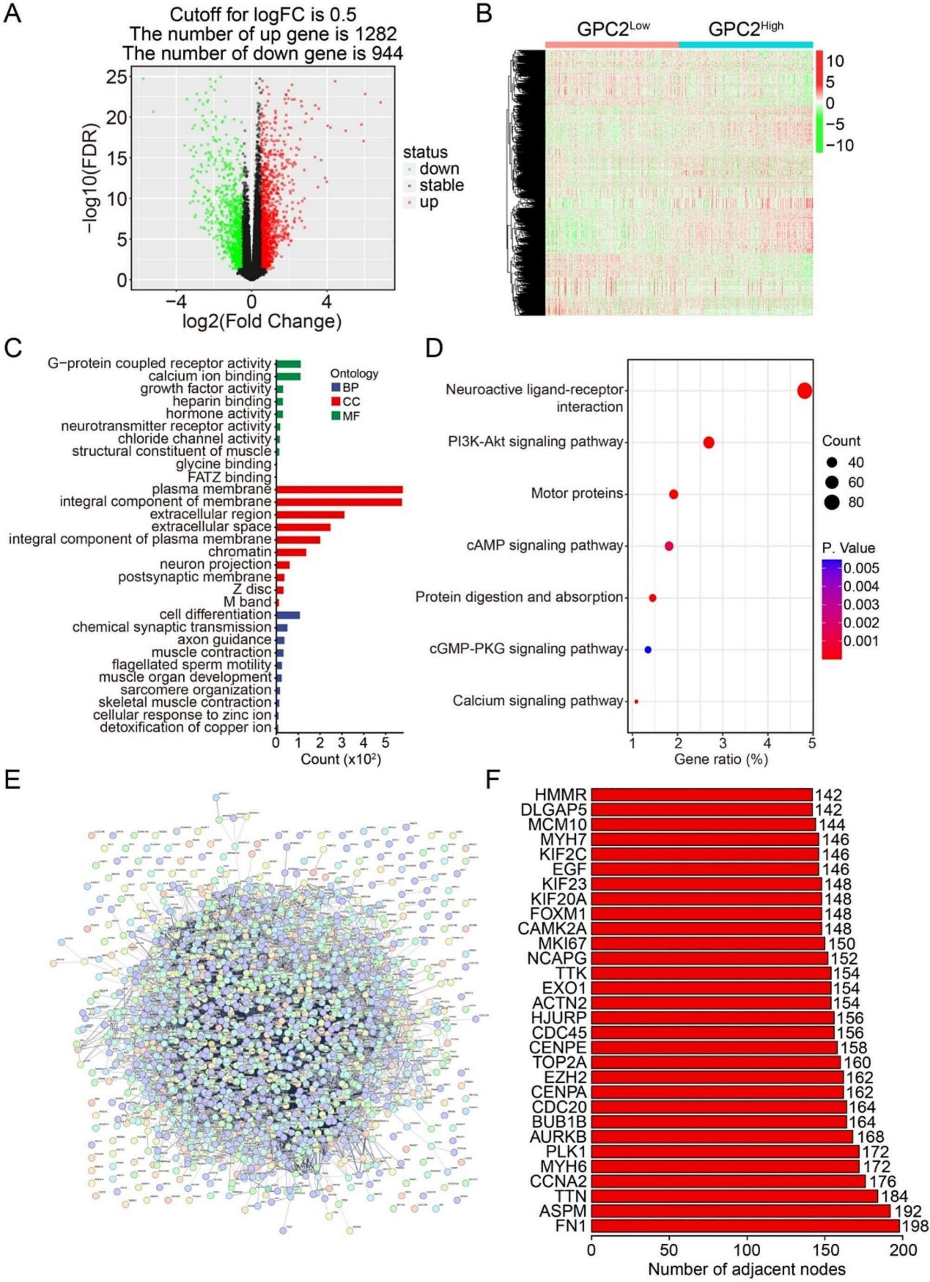
Bioinformatic analysis identified GPC2-related DEGs and functional enrichment analysis. (**A**) Volcano plot showing the differentially expressed genes between GPC2^High^ and GPC2^Low^ group. The criteria of DEG were set as |log2(fold change) |>0.5 and FDR < 0.05. (**B**) Heatmap showing the expression profiles of GPC2-related DEGs in prostate cancer tissues of TCGA database. (**C**) GO enrichment analysis of GPC2-related DEGs. (**D**) KEGG enrichment analysis of GPC2-related DEGs showing PI3K/AKT signaling pathway was markedly enriched. (**E**) Construction of a PPI network using the GPC2-related DEGs. (**F**) The top thirty ranking genes in the PPI network

We next explored the molecular mechanism by which GPC2 promoted cell proliferation, migration, and invasion. The TCGA prostate carcinoma dataset was separated into GPC2^High^ and GPC2^Low^ groups based on the median expression level of GPC2, the differentially expressed genes (DEGs) among the two groups were identified using bioinformatic method. As shown in Fig. 4A, GPC2^High^ group exhibited 944 downregulated and 1282 upregulated genes compared to GPC2^Low^ group using a threshold of log2|Fold change|>0.5 and FDR < 0.05 as the cutoff for a significantly altered gene, and these DEGs were regarded as GPC2-related DEGs. The expression profile of these GPC2-related DEGs in TCGA prostate carcinoma was displayed in Fig. 4B. GO enrichment analysis indicated that these GPC2-related DEGs were particularly enriched in cell differentiation, chemical synaptic transmission, and axon guidance in terms of biological process. As for the cellular component, plasma membrane, integral component of membrane, and extracellular region were the three most enriched terms. With respect to molecular function, these GPC2-related DEGs were mainly enriched in G-protein coupled receptor activity, calcium ion binding, and growth factor activity (Fig. 4C). KEGG enrichment analysis delineated that neuroactive ligand-receptor interaction, PI3K-Akt signaling pathway, cAMP signaling pathway, cGMP-PKG signaling pathway, and calcium signaling pathway were significantly enriched, suggesting a potential regulatory role of GPC2 on these pathways (Fig. 4D). Moreover, we constructed a protein-protein interaction network using these GPC2-related DEGs (Fig. 4E), and the top three ranking genes in the PPI network were FN1, ASPM, and TTN (Fig. 4F).

### GPC2 positively regulated PI3K/AKT signaling pathway through MDK

**Fig. 5:**
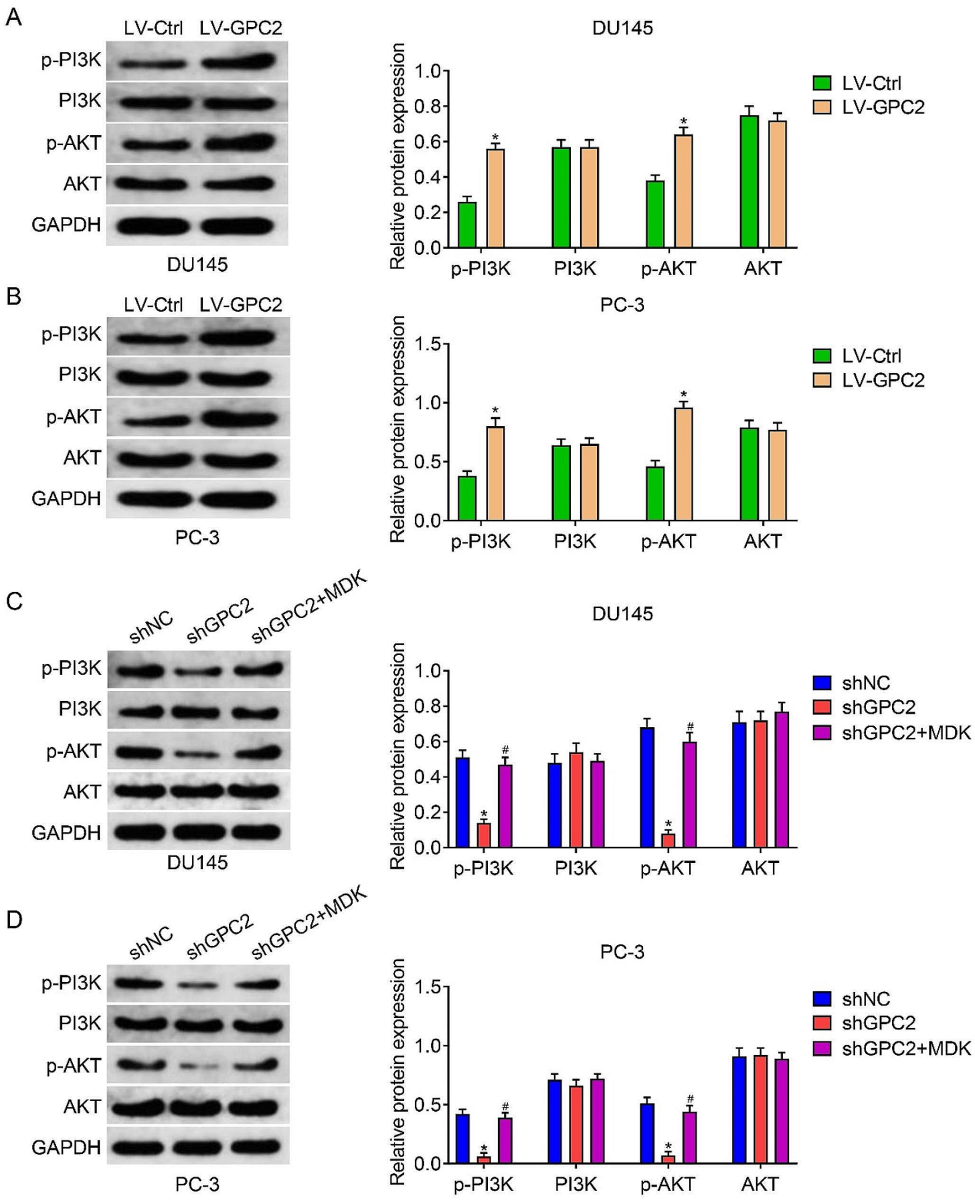
GPC2 positively regulated PI3K/AKT signaling pathway through MDK. (**A-B**) Western blot assay was performed to detect the phosphorylation of PI3K and AKT in LV-Ctrl and LV-GPC2 groups of prostate cancer cells, and quantitative analyses. (**C-D**) The effect of MDK overexpression on the protein levels of PI3K, p-PI3K, AKT, and p-AKT in prostate cancer cells after knocking down of GPC2, and quantitative analyses. *Indicates a significant difference between the shGPC2 and shNC groups. ^#^Indicates a significant difference between the shGPC2 + MDK and shGPC2 groups. *P < 0.05. ^#^*P* < 0.05

Among the aforementioned enriched pathways of the KEGG analysis, we became particularly interested in PI3K/AKT signaling pathway, as it is a critical pathway during carcinogenesis and tumor progression. Therefore, we further assessed whether GPC2 regulated PI3K/AKT signaling pathway by detecting the protein levels of PI3K, p-PI3K, AKT, and p-AKT. Western blot assay showed that overexpression of GPC2 increased the phosphorylation of PI3K and AKT, while had no effect on the total protein levels of PI3K and AKT (Fig. 5A-B), suggesting that GPC2 positively regulated the activation of PI3K/AKT signaling pathway. Then, we asked by which means that GPC2 regulated PI3K/AKT signaling pathway. By mining the STRING database and previous researches, we found that MDK, a growth factor that induced the activation of a series of downstream signal cascades, might be a direct target of GPC2 since there was interaction between GPC2 and MDK, and MDK might be participated in the activation of PI3K/AKT signaling pathway(.Han et al. 2023; Hu et al. 2021; Kurosawa et al. 2001). Immunoprecipitation assay confirmed the physical interaction between GPC2 and MDK (Supplementary Fig. S1A). Then, we manipulated the expression of MDK through plasmid transfection in prostate cancer cell lines that were stably knocking down of GPC2, and further measured the protein levels of PI3K, p-PI3K, AKT, as well as p-AKT. The transfection efficiency of MDK overexpression plasmid was confirmed via qRT-PCR assay (Supplementary Fig. S1B). Western blot assays revealed that knockdown of GPC2 reduced the expression of MDK, p-PI3K and p-AKT (Fig. 5C-D and Supplementary Fig. S2A-B), further demonstrating the GPC2 participated in the regulation of PI3K/AKT signaling pathway(.Filippou et al. 2020). Moreover, overexpression of MDK could attenuate GPC2 knockdown induced downregulation of p-PI3K and p-AKT (Fig. 5C-D). Taken together, the results we have presented so far indicated that GPC2 could positively regulate the activation of PI3K/AKT signaling pathway at least partly through MDK.

### Overexpression of MDK reversed the inhibitory effect of GPC2 knockdown on cell proliferation, migration, and invasion in prostate cancer

**Fig. 6:**
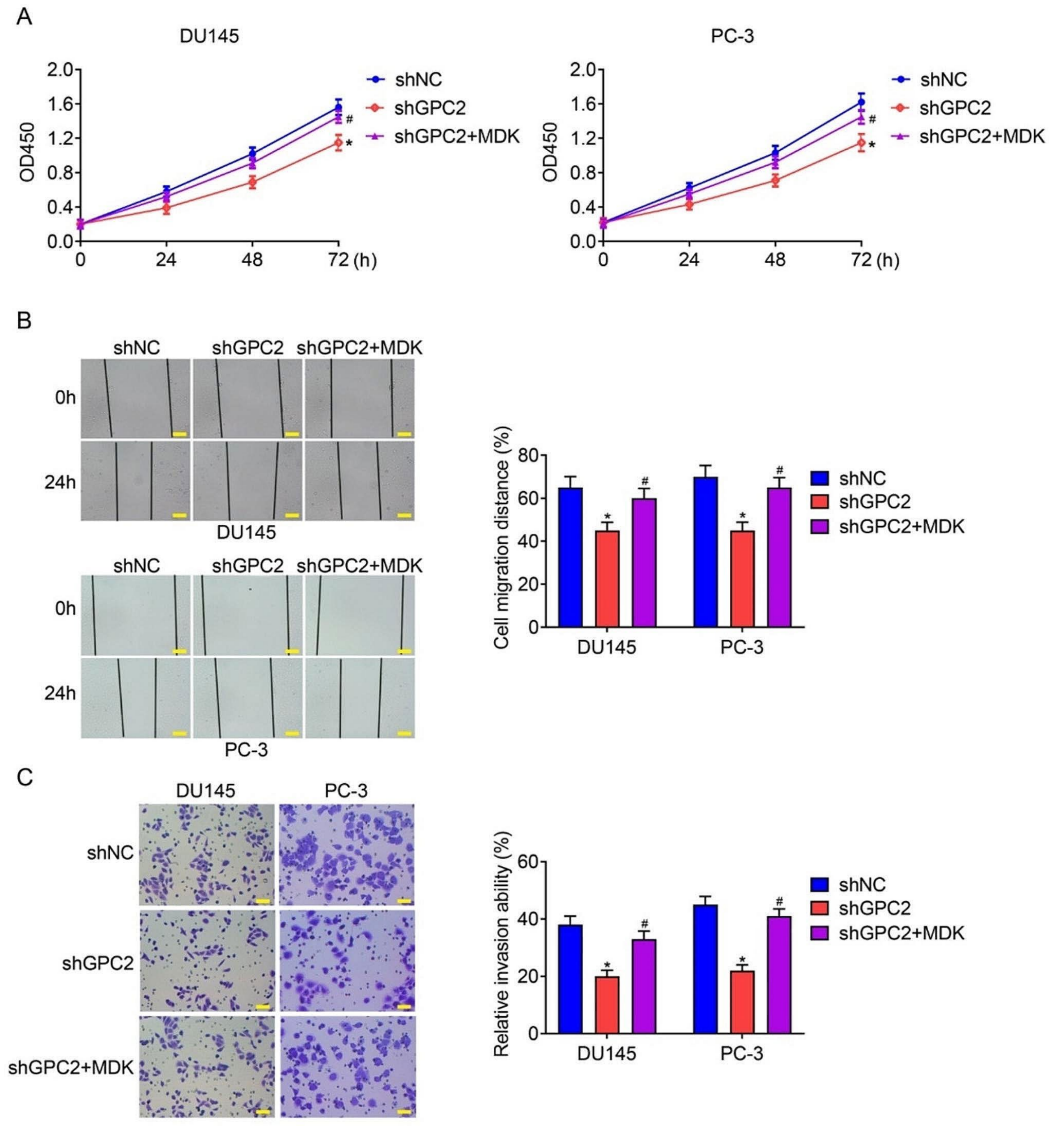
Overexpression of MDK reversed the inhibitory effect of GPC2 knockdown on cell proliferation, migration, and invasion in prostate cancer. (**A**) CCK-8 assay was conducted to assess cell proliferation ability in prostate cancer cells transfected with shNC, shGPC2, and shGPC2 + MDK. (**B-C**) Would healing and transwell invasion assays were performed to detect cell migration and invasion abilities in prostate cancer cells transfected with shNC, shGPC2, and shGPC2 + MDK. *Indicates a significant difference between the shGPC2 and shNC groups. ^#^Indicates a significant difference between the shGPC2 + MDK and shGPC2 groups. *P < 0.05. ^#^*P* < 0.05

Finally, to investigate whether GPC2 knockdown inhibited the malignancy of prostate cancer via MDK, we upregulated the expression of MDK in DU145 and PC-3 cells that were stably knocking down of GPC2, and performed functional assays. CCK-8 results showed that overexpression of MDK could partly attenuated GPC2 knockdown induced decreased cell proliferation ability (Fig. 6A). Wound healing and transwell invasion assays revealed that knock down of GPC2 impaired the abilities of cell migration and invasion, however, increasing the expression of MDK could reverse this phenomenon (Fig. 6B-C). Take together, our results indicated that overexpression of MDK could reverse the inhibitory effect of GPC2 knockdown on malignancy of prostate cancer.

## Discussion

The genes of the glypican family are highly conserved across animal species and play important roles in fundamental biological processes such cell survival, motility, and differentiation(Dwivedi et al. 2013; Kamimura and Maeda 2021). Therefore, it is not surprising that change in the expression of glypican genes has been reported in multiple human cancers(.McGough et al. 2020; Melo et al. 2015; Zhou et al. 2018). Recently, much attention had been paid to the role of GPC2 in tumors. GPC2 was shown to be highly expressed in colon adenocarcinoma (COAD) and was associated with advanced tumor stage and poor prognosis. GPC2 deficiency induced cell cycle arrest and apoptosis in COAD cell lines, as well as inhibiting cell proliferation, migration, and invasion(.Lin, He, Ni, Zhang, Liu, Mao, Huang and Lin 2022). In early-stage pancreatic ductal adenocarcinoma (PDAC), GPC2 expression was higher in tumor tissues when compared to adjacent normal tissues. Moreover, survival analysis based on TCGA database revealed that higher expression of GPC2 was correlated with unfavorable survival in PDAC(.Liu et al. 2020). In neuroblastoma (NB), GPC2 was reported to be upregulated in tumor tissues and was selectively expressed on the cell surface. More importantly, GPC2 was minimally expressed in normal tissues, making it an attractive target for immunotherapy of neuroblastoma. Chimeric antigen receptor (CAR) T cells targeting GPC2 showed excellent tumor inhibitory role in neuroblastoma in *vivo*(.Li et al. 2021). GPC2 antibody-drug conjugate could change the tumor microenvironment (TME) to a proinflammatory state in neuroblastoma, and might promote immunogenic cell death and enhance antitumor immune response(.Pascual-Pasto et al. 2022). In another report, Raman and his colleague constructed a GPC2-directed antibody-drug conjugate (ADC). Further experiments confirmed that this ADC could inhibit neuroblastoma and small-cell lung cancers tumor regression, which was associated with DNA damage, cell apoptosis, and bystander cell killing(.Raman, Buongervino, Lane, Zhelev, Zhu, Cui, Martinez, Martinez, Wang, Upton, Patel, Rathi, Navia, Harmon, Li, Pawel, Dimitrov, Maris, Julien and Bosse 2021). There studies indicated that GPC2 might be an ideal therapeutic target in cancer, while GPC2 role in prostate cancer remains unclear.

In this work, our findings revealed that GPC2, which was higher in prostate cancer than that in adjacent normal tissue, was positively correlated with clinical stage and lymphatic metastasis in prostate cancer. Kaplan-Meier survival analysis indicated that higher expression of GPC2 predicted worse clinical outcome in patients with prostate cancer, suggesting a potential role of GPC2 in prognostic prediction in prostate cancer. A real-world cohort of prostate cancer patients needs to be collected to further test the predictive ability of GPC2 in prostate cancer. To explore the functional role of GPC2 on malignancy of PC cells, we performed in *vitro* experiments to examine the effect of GPC2 knockdown or overexpression on cell proliferation, migration, and invasion. Our results indicated that GPC2 served as an oncoprotein in prostate cancer, and the knockdown of GPC2 impaired the abilities of cell proliferation, migration, and invasion, while overexpression of GPC2 had the opposite effect. Taken together, these in *vitro* experiment results that we have presented so far indicated that GPC2 might be a potential therapeutic target in prostate cancer, further exploration should be conducted in *vivo* to confirm the anti-tumor efficiency of GPC2-based target therapy.

To investigate the potential molecular mechanism underlying the effect of GPC2 on cell proliferation, migration, and invasion, we employed bioinformatic analysis on the gene expression matrix of the prostate cancer tissues in TCGA database. We identified GPC2-related DEGs by separating the gene expression matrix into GPC2^High^ and GPC2^Low^ groups and further KEGG functional enrichment analysis revealed that these GPC2-related DEGs were particularly enriched in PI3K/AKT pathway, which attracted our attention due to its vital role in prostate carcinogenesis and progression(.Chen et al. 2016). The phosphatidylinositol 3-kinase (PI3K), comprised by three subunits, the regulatory subunits p85 and p55, and the catalytic subunit p110, functions as a plasma membrane-associated protein kinase(.Toren and Zoubeidi 2014). Once activation by the upstream receptor tyrosine kinases (RTKs) or non-RTKs, PI3K phosphorylates the PIP2 to produce PIP3, which further activates intracellular signaling by recruiting pleckstrin homology (PH) domain-containing proteins, including AKT, to the cell membrane. Then, the activated AKT proteins were translocated into cytoplasm and nucleus, and leading to the activation of downstream targets that participated in regulating cancer survival, angiogenesis, and metastasis(.Alzahrani 2019; Chen, Zhou, Wu, Li, Wen, Sha and Wen 2016, He et al. 2021; Karar and Maity 2011; Yang et al. 2019). To detect whether there were association between GPC2 and PI3K/AKT signaling, we examined the expression level of PI3K, p-PI3K, AKT, and p-AKT in prostate cancer cells by western blot assay. As expected, our results showed that knockdown of GPC2 resulted in decreased expression of p-PI3K and p-AKT, while GPC2 overexpression had the opposite effect, suggesting that GPC2 positively regulated the activation of PI3K/AKT signaling pathway.

Then, we asked by which means that GPC2 regulated PI3K/AKT signaling pathway. We first analyzed the interacting proteins of GPC2 through STRING database and found that MDK might be a direct target of GPC2. Midkine (MDK), encoded by *MDK* gene, was a multifunctional heparin-binding protein that could be secreted into the blood, urinary, and cerebrospinal fluid(.Cerezo-Wallis et al. 2020; Filippou et al. 2020; Muramatsu and Kadomatsu 2014). Recently, researches on MDK had escalated, especially in the field of cancer. MDK was reported to be upregulated in multiple cancers than that in healthy individuals, and this aberrant expression was associated with tumor progression and patients’ prognosis(.Lu et al. 2018; Xia et al. 2022). Functionally, MDK could be served as critical regulator of malignant behaviors of cancers, including cell proliferation, survival, metastasis, angiogenesis, stemness, and chemoresistance by various pathways(.Donia and Jönsson 2021; Kishida and Kadomatsu 2014; Kishida et al. 2013; Tang et al. 2022). Of note, the PI3K/AKT signaling was one of the most important downstream pathways of MDK that exerted roles in cancer. For example, Hu et al.(.Hu, Qin, Li, Wei, Mo, Fan, Lei, Wei and Zou 2021) reported that MDK promoted glioblastoma cell proliferation, migration, and invasion via activating the PI3K/AKT signaling. In breast cancer, MDK was identified as a direct downstream protein of miR-1275. Silence of miR-1275 leads to upregulation of MDK and further activating PI3K/AKT signaling to enhance the properties of cancer stem cells and promote chemoresistance(.Han, Li, Xu, Fu, Wang, Wang, Xia, Wang and Ma 2023). To explore whether GPC2 promotes the activation of PI3K/AKT signaling via MDK in prostate cancer, we first confirmed the physical interaction between GPC2 and MDK through immunoprecipitation assay. Then, we manipulated the expression of MDK after knocking down of GPC2 and detected the protein levels of p-PI3K and p-AKT. Our results revealed that overexpression of MDK could attenuate GPC2 knockdown induced downregulation of p-PI3K and p-AKT, therefore suggesting that GPC2 positively regulated PI3K/AKT signaling pathway at least partly through MDK. Moreover, rescue experiments showed that overexpression of MDK could attenuate the inhibitory effect of GPC2 knockdown on prostate cancer proliferation, migration, and invasion. To simplify, our research reveals that GPC2 promotes prostate cancer progression via MDK-mediated activation of PI3K/AKT signaling pathway, and GPC2 might be a potential therapeutic target in prostate cancer.

Our study had some limitations. First, GPC2 expression in prostate cancer tissues should also be examined by experiments to make our result more convincing. Second, in *vivo* experiments needed to be performed to investigate the inhibitory effect of GPC2-based target therapy in prostate cancer.

In summary, our study identified GPC2 as an oncoprotein in prostate cancer. GPC2 was upregulated in tumor tissues and correlated with clinical stage, tumor metastasis, and patients’ prognosis. GPC2 promotes prostate cancer cell proliferation, migration, and invasion via MDK-mediated activation of PI3K/AKT signaling pathway. GPC2 might be a promising prognosis predictor and potential therapeutic target.

### Electronic supplementary material

Below is the link to the electronic supplementary material.

Supplementary Material 1

## Data Availability

No datasets were generated or analysed during the current study.

## References

[CR1] Alzahrani AS (2019) PI3K/Akt/mTOR inhibitors in cancer: at the bench and bedside. Semin Cancer Biol 59:125–132. 10.1016/j.semcancer.2019.07.009 31323288 10.1016/j.semcancer.2019.07.009

[CR2] Bosse KR, Raman P, Zhu Z, Lane M, Martinez D, Heitzeneder S, Rathi KS, Kendsersky NM, Randall M, Donovan L, Morrissy S, Sussman RT, Zhelev DV, Feng Y, Wang Y, Hwang J, Lopez G, Harenza JL, Wei JS, Pawel B, Bhatti T, Santi M, Ganguly A, Khan J, Marra MA, Taylor MD, Dimitrov DS, Mackall CL, Maris JM (2017) Identification of GPC2 as an oncoprotein and candidate immunotherapeutic target in High-Risk Neuroblastoma. Cancer Cell 32:295–309e212. 10.1016/j.ccell.2017.08.003 28898695 10.1016/j.ccell.2017.08.003 PMC5600520

[CR3] Cerezo-Wallis D, Contreras-Alcalde M, Troulé K, Catena X, Mucientes C, Calvo TG, Cañón E, Tejedo C, Pennacchi PC, Hogan S, Kölblinger P, Tejero H, Chen AX, Ibarz N, Graña-Castro O, Martinez L, Muñoz J, Ortiz-Romero P, Rodriguez-Peralto JL, Gómez-López G, Al-Shahrour F, Rabadán R, Levesque MP, Olmeda D, Soengas MS (2020) Midkine rewires the melanoma microenvironment toward a tolerogenic and immune-resistant state. Nat Med 26:1865–1877. 10.1038/s41591-020-1073-3 33077955 10.1038/s41591-020-1073-3

[CR5] Chen H, Zhou L, Wu X, Li R, Wen J, Sha J, Wen X (2016) The PI3K/AKT pathway in the pathogenesis of prostate cancer. Front Biosci (Landmark Ed) 21:1084–1091. 10.2741/4443 27100493 10.2741/4443

[CR4] Chen G, Luo D, Zhong N, Li D, Zheng J, Liao H, Li Z, Lin X, Chen Q, Zhang C, Lu Y, Chan YT, Ren Q, Wang N, Feng Y (2022) GPC2 is a potential Diagnostic, Immunological, and Prognostic Biomarker in Pan-cancer. Front Immunol 13:857308. 10.3389/fimmu.2022.857308 35345673 10.3389/fimmu.2022.857308 PMC8957202

[CR6] Desai K, McManus JM, Sharifi N (2021) Hormonal therapy for prostate Cancer. Endocr Rev 42:354–373. 10.1210/endrev/bnab002 33480983 10.1210/endrev/bnab002 PMC8152444

[CR7] Donia M, Jönsson G (2021) Midkine-A potential therapeutic target in melanoma. Pigment Cell Melanoma Res 34:834–835. 10.1111/pcmr.12967 33599989 10.1111/pcmr.12967

[CR8] Dwivedi PP, Lam N, Powell BC (2013) Boning up on glypicans–opportunities for new insights into bone biology. Cell Biochem Funct 31:91–114. 10.1002/cbf.2939 23297043 10.1002/cbf.2939

[CR9] Filippou PS, Karagiannis GS, Constantinidou A (2020) Midkine (MDK) growth factor: a key player in cancer progression and a promising therapeutic target. Oncogene 39:2040–2054. 10.1038/s41388-019-1124-8 31801970 10.1038/s41388-019-1124-8

[CR10] Filmus J (2022) The function of glypicans in the mammalian embryo. Am J Physiol Cell Physiol 322:C694–c698. 10.1152/ajpcell.00045.2022 35235423 10.1152/ajpcell.00045.2022 PMC9722250

[CR11] Filmus J, Capurro M (2014) The role of glypicans in hedgehog signaling. Matrix Biol 35:248–252. 10.1016/j.matbio.2013.12.007 24412155 10.1016/j.matbio.2013.12.007

[CR12] Haffner MC, Zwart W, Roudier MP, True LD, Nelson WG, Epstein JI, De Marzo AM, Nelson PS, Yegnasubramanian S (2021) Genomic and phenotypic heterogeneity in prostate cancer. Nat Rev Urol 18:79–92. 10.1038/s41585-020-00400-w 33328650 10.1038/s41585-020-00400-w PMC7969494

[CR13] Han X, Li M, Xu J, Fu J, Wang X, Wang J, Xia T, Wang S, Ma G (2023) miR-1275 targets MDK/AKT signaling to inhibit breast cancer chemoresistance by lessening the properties of cancer stem cells. Int J Biol Sci 19:89–103. 10.7150/ijbs.74227 36594100 10.7150/ijbs.74227 PMC9760432

[CR14] He Y, Sun MM, Zhang GG, Yang J, Chen KS, Xu WW, Li B (2021) Targeting PI3K/Akt signal transduction for cancer therapy. Signal Transduct Target Ther 6:425. 10.1038/s41392-021-00828-5 34916492 10.1038/s41392-021-00828-5 PMC8677728

[CR15] Hu B, Qin C, Li L, Wei L, Mo X, Fan H, Lei Y, Wei F, Zou D (2021) Midkine promotes glioblastoma progression via PI3K-Akt signaling. Cancer Cell Int 21:509. 10.1186/s12935-021-02212-3 34556138 10.1186/s12935-021-02212-3 PMC8461913

[CR16] Kamimura K, Maeda N (2021) Glypicans and Heparan Sulfate in synaptic development, neural plasticity, and neurological disorders. Front Neural Circuits 15:595596. 10.3389/fncir.2021.595596 33679334 10.3389/fncir.2021.595596 PMC7928303

[CR17] Karar J, Maity A (2011) PI3K/AKT/mTOR pathway in Angiogenesis. Front Mol Neurosci 4:51. 10.3389/fnmol.2011.00051 22144946 10.3389/fnmol.2011.00051 PMC3228996

[CR18] Kaur SP, Cummings BS (2019) Role of glypicans in regulation of the tumor microenvironment and cancer progression. Biochem Pharmacol 168:108–118. 10.1016/j.bcp.2019.06.020 31251939 10.1016/j.bcp.2019.06.020

[CR19] Kishida S, Kadomatsu K (2014) Involvement of midkine in neuroblastoma tumourigenesis. Br J Pharmacol 171:896–904. 10.1111/bph.12442 24116381 10.1111/bph.12442 PMC3925028

[CR20] Kishida S, Mu P, Miyakawa S, Fujiwara M, Abe T, Sakamoto K, Onishi A, Nakamura Y, Kadomatsu K (2013) Midkine promotes neuroblastoma through Notch2 signaling. Cancer Res 73:1318–1327. 10.1158/0008-5472.Can-12-3070 23243020 10.1158/0008-5472.Can-12-3070

[CR21] Kurosawa N, Chen GY, Kadomatsu K, Ikematsu S, Sakuma S, Muramatsu T (2001) Glypican-2 binds to midkine: the role of glypican-2 in neuronal cell adhesion and neurite outgrowth. Glycoconj J 18:499–507. 10.1023/a:1016042303253 12084985 10.1023/a:1016042303253

[CR22] Li N, Gao W, Zhang YF, Ho M (2018) Glypicans as Cancer therapeutic targets. Trends Cancer 4:741–754. 10.1016/j.trecan.2018.09.004 30352677 10.1016/j.trecan.2018.09.004 PMC6209326

[CR23] Li N, Spetz MR, Ho M (2020) The role of glypicans in Cancer Progression and Therapy. J Histochem Cytochem 68:841–862. 10.1369/0022155420933709 32623934 10.1369/0022155420933709 PMC7711243

[CR24] Li N, Torres MB, Spetz MR, Wang R, Peng L, Tian M, Dower CM, Nguyen R, Sun M, Tai CH, de Val N, Cachau R, Wu X, Hewitt SM, Kaplan RN, Khan J, St Croix B, Thiele CJ, Ho M (2021) CAR T cells targeting tumor-associated exons of glypican 2 regress neuroblastoma in mice. Cell Rep Med 2:100297. 10.1016/j.xcrm.2021.100297 34195677 10.1016/j.xcrm.2021.100297 PMC8233664

[CR25] Lin L, He Y, Ni Z, Zhang M, Liu J, Mao Q, Huang B, Lin J (2022) GPC2 deficiency inhibits cell growth and metastasis in colon adenocarcinoma. Open Med (Wars) 17:304–316. 10.1515/med-2022-0421 35233466 10.1515/med-2022-0421 PMC8847712

[CR26] Liu JQ, Liao XW, Wang XK, Yang CK, Zhou X, Liu ZQ, Han QF, Fu TH, Zhu GZ, Han CY, Su H, Huang JL, Ruan GT, Yan L, Ye XP, Peng T (2020) Prognostic value of Glypican family genes in early-stage pancreatic ductal adenocarcinoma after pancreaticoduodenectomy and possible mechanisms. BMC Gastroenterol 20:415. 10.1186/s12876-020-01560-0 33302876 10.1186/s12876-020-01560-0 PMC7731467

[CR27] Lu J, Liu QH, Wang F, Tan JJ, Deng YQ, Peng XH, Liu X, Zhang B, Xu X, Li XP (2018) Exosomal miR-9 inhibits angiogenesis by targeting MDK and regulating PDK/AKT pathway in nasopharyngeal carcinoma. J Exp Clin Cancer Res 37:147. 10.1186/s13046-018-0814-3 30001734 10.1186/s13046-018-0814-3 PMC6044044

[CR28] McGough IJ, Vecchia L, Bishop B, Malinauskas T, Beckett K, Joshi D, O’Reilly N, Siebold C, Jones EY, Vincent JP (2020) Glypicans shield the wnt lipid moiety to enable signalling at a distance. Nature 585:85–90. 10.1038/s41586-020-2498-z 32699409 10.1038/s41586-020-2498-z PMC7610841

[CR29] Melo SA, Luecke LB, Kahlert C, Fernandez AF, Gammon ST, Kaye J, LeBleu VS, Mittendorf EA, Weitz J, Rahbari N, Reissfelder C, Pilarsky C, Fraga MF, Piwnica-Worms D, Kalluri R (2015) Glypican-1 identifies cancer exosomes and detects early pancreatic cancer. Nature 523:177–182. 10.1038/nature14581 26106858 10.1038/nature14581 PMC4825698

[CR30] Muramatsu T, Kadomatsu K (2014) Midkine: an emerging target of drug development for treatment of multiple diseases. Br J Pharmacol 171:811–813. 10.1111/bph.12571 24460672 10.1111/bph.12571 PMC3925019

[CR31] Pascual-Pasto G, McIntyre B, Shraim R, Buongervino SN, Erbe AK, Zhelev DV, Sadirova S, Giudice AM, Martinez D, Garcia-Gerique L, Dimitrov DS, Sondel PM, Bosse KR (2022) GPC2 antibody-drug conjugate reprograms the neuroblastoma immune milieu to enhance macrophage-driven therapies. J Immunother Cancer 10. 10.1136/jitc-2022-004704 10.1136/jitc-2022-004704 PMC9723962 36460335

[CR32] Raman S, Buongervino SN, Lane MV, Zhelev DV, Zhu Z, Cui H, Martinez B, Martinez D, Wang Y, Upton K, Patel K, Rathi KS, Navia CT, Harmon DB, Li Y, Pawel B, Dimitrov DS, Maris JM, Julien JP, Bosse KR (2021) A GPC2 antibody-drug conjugate is efficacious against neuroblastoma and small-cell lung cancer via binding a conformational epitope. Cell Rep Med 2:100344. 10.1016/j.xcrm.2021.100344 34337560 10.1016/j.xcrm.2021.100344 PMC8324494

[CR33] Rebello RJ, Oing C, Knudsen KE, Loeb S, Johnson DC, Reiter RE, Gillessen S, Van der Kwast T, Bristow RG (2021) Prostate cancer. Nat Rev Dis Primers 7:9. 10.1038/s41572-020-00243-0 33542230 10.1038/s41572-020-00243-0

[CR34] Sekhoacha M, Riet K, Motloung P, Gumenku L, Adegoke A, Mashele S (2022) Prostate Cancer Review: Genetics, diagnosis, Treatment options, and alternative approaches. Molecules 27. 10.3390/molecules27175730 10.3390/molecules27175730 PMC9457814 36080493

[CR35] Siegel RL, Miller KD, Jemal A (2020) Cancer statistics, 2020. CA Cancer J Clin 70:7–30. 10.3322/caac.21590 31912902 10.3322/caac.21590

[CR36] Tang Y, Kwiatkowski DJ, Henske EP (2022) Midkine expression by stem-like tumor cells drives persistence to mTOR inhibition and an immune-suppressive microenvironment. Nat Commun 13:5018. 10.1038/s41467-022-32673-7 36028490 10.1038/s41467-022-32673-7 PMC9418323

[CR37] Toren P, Zoubeidi A (2014) Targeting the PI3K/Akt pathway in prostate cancer: challenges and opportunities (review). Int J Oncol 45:1793–1801. 10.3892/ijo.2014.2601 25120209 10.3892/ijo.2014.2601

[CR38] Wang G, Zhao D, Spring DJ, DePinho RA (2018) Genetics and biology of prostate cancer. Genes Dev 32:1105–1140. 10.1101/gad.315739.118 30181359 10.1101/gad.315739.118 PMC6120714

[CR39] Wasim S, Lee SY, Kim J (2022) Complexities of prostate Cancer. Int J Mol Sci 23. 10.3390/ijms232214257 10.3390/ijms232214257 PMC9696501 36430730

[CR40] Xia T, Chen D, Liu X, Qi H, Wang W, Chen H, Ling T, Otkur W, Zhang CS, Kim J, Lin SC, Piao HL (2022) Midkine noncanonically suppresses AMPK activation through disrupting the LKB1-STRAD-Mo25 complex. Cell Death Dis 13:414. 10.1038/s41419-022-04801-0 35487917 10.1038/s41419-022-04801-0 PMC9054788

[CR41] Yamada Y, Beltran H (2021) The treatment landscape of metastatic prostate cancer. Cancer Lett 519:20–29. 10.1016/j.canlet.2021.06.010 34153403 10.1016/j.canlet.2021.06.010 PMC8403655

[CR42] Yang Q, Jiang W, Hou P (2019) Emerging role of PI3K/AKT in tumor-related epigenetic regulation. Semin Cancer Biol 59:112–124. 10.1016/j.semcancer.2019.04.001 30951826 10.1016/j.semcancer.2019.04.001

[CR43] Zhou F, Shang W, Yu X, Tian J (2018) Glypican-3: a promising biomarker for hepatocellular carcinoma diagnosis and treatment. Med Res Rev 38:741–767. 10.1002/med.21455 28621802 10.1002/med.21455

