# Supplementary material for: GPC2 promotes prostate cancer progression via MDK-mediated activation of PI3K/AKT signaling pathway
Source: Funct Integr Genomics. 2024 Jul 17;24(4):127. doi: 10.1007/s10142-024-01406-y (PMC11252201; doi:10.1007/s10142-024-01406-y)
Supplement: Supplementary file 1 — Supplementary Material 1 [file 10142_2024_1406_MOESM1_ESM.docx]

**Supplementary Fig. 1**. **MDK was a direct target of GPC2.** (A) Immunoprecipitation assay confirmed the interaction of GPC2 and MDK. (B) qRT-PCR was performed to confirm the overexpression efficiency of MDK. **P* < 0.05.


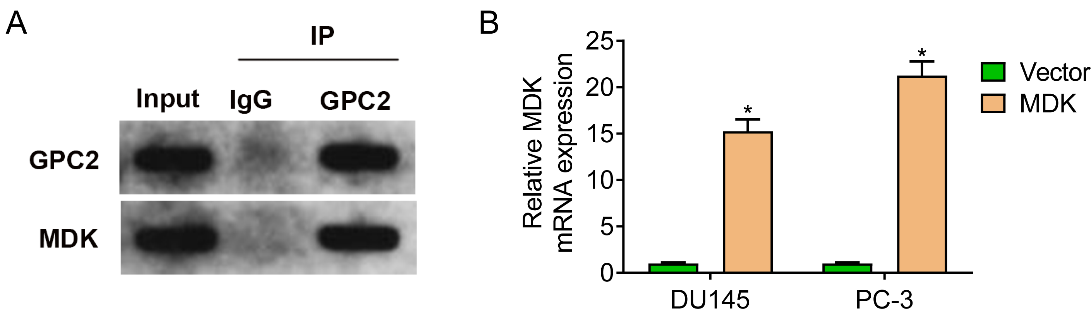


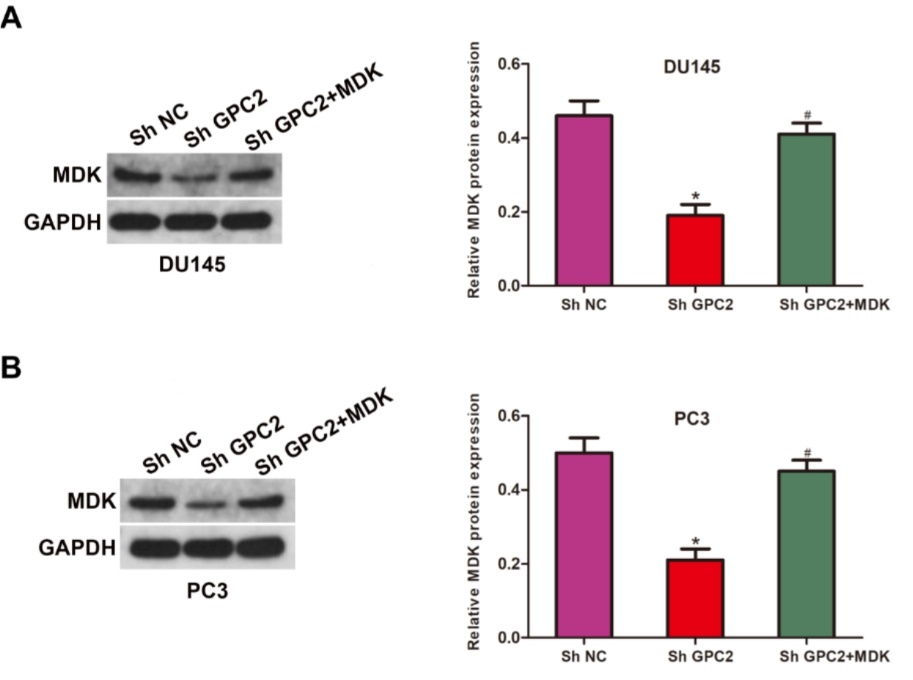


**Supplementary Fig. 2**. Western blot analysis of MDK protein levels in DU145 (A) and PC-3 cells (B). *Indicates a significant difference between the shGPC2 and shNC groups. ^#^Indicates a significant difference between the shGPC2 + MDK and shGPC2 groups. **P* < 0.05. ^#^*P* < 0.05.

**Supplementary Table 1. The detailed information of BCa patients.**

| **Characteristics** | **Number of cases** |
| --- | --- |
| **Age (y)** |  |
| < 65 | 2 |
| ≥ 65 | 8 |
| **Preoperative PSA (ng/mL)** |  |
| ≤ 10 | 4 |
| > 10 | 6 |
| **Gleason score** |  |
| ≤ 7 | 7 |
| > 7 | 3 |
| **Clinical stage** |  |
| T1 + T2 | 6 |
| T3 + T4 | 4 |
| **Lymph node metastasis** |  |
| Without | 5 |
| With | 5 |
| **Bone metastasis** |  |
| Without | 8 |
| With | 2 |
